# Effects of Different Dietary Interventions on Calcitriol, Parathyroid Hormone, Calcium, and Phosphorus: Results from the DASH Trial

**DOI:** 10.3390/nu10030367

**Published:** 2018-03-17

**Authors:** Ahmed Hassoon, Erin D. Michos, Edgar R. Miller, Zeni Crisp, Lawrence J. Appel

**Affiliations:** 1 Department of Epidemiology, Johns Hopkins Bloomberg School of Public Health, , 615 N. Wolfe Street, Baltimore, MD 21205, USA; edonnell@jhmi.edu (E.D.M.); ermiller@jhmi.edu (E.R.M.); lappel@jhmi.edu (L.J.A.); 2 Paul L. Foster School of Medicine, Texas Tech University Health Sciences Center, 5001 El Paso Drive, El Paso, TX 79905, USA; zeni.crisp@ttuhsc.edu

**Keywords:** DASH trial, calcitriol, parathyroid hormone, vitamin D

## Abstract

The “Dietary Approaches to Stop Hypertension” (DASH) diet, rich in fiber and low-fat dairy, effectively lowers blood pressure. DASH’s effect on calcitriol and other markers of bone-mineral metabolism is unknown. This secondary analysis of the DASH trial aimed to determine the effect of dietary patterns on blood concentrations of calcitriol, parathyroid hormone (PTH), ionized calcium, and urinary excretion of calcium and phosphorus. Outcomes were available in 334 participants in the trial. After a 3-week run-in on the control diet, participants were randomized to control, fruits and vegetables (F&V), or DASH diets. Outcomes were assessed at the end of run-in, and during the last week of the intervention period. Mean age of participants was 45.7 ± 10.7 years, 46% female, and 57% African-American. Mean ± Standard Deviation(SD) baseline serum concentrations of calcitriol, PTH, and ionized calcium were 37.8 ± 9.2 pg/mL, 46.1 ± 18.5 pg/mL and 5.2 ± 0.23 mg/dL, respectively. Mean (±SD) urinary calcium and phosphorus excretions were 150.1 ± 77.8 and 708.0 ± 251.8 mg/24 h, respectively. Compared with control, DASH reduced calcitriol −3.32 pg/mL (*p* = 0.004). Otherwise, there was no significant effect on other biomarkers. DASH lowered serum calcitriol perhaps more among African-Americans. These results raise important questions about the interpretation and clinical significance of low calcitriol concentrations in the setting of recommended diets.

## 1. Introduction

Interest in the relationship of bone-mineral metabolism with cardiovascular health is burgeoning. Calcidiol (25-hydroxyvitamin D) is the primary circulating form of vitamin D and is metabolized by the kidney into calcitriol (1,25-dihydroxyvitamin D3), the biologically active form of vitamin D [1]. Calcitriol affects bone mineral metabolism [2], but studies have shown that calcitriol might also affect cardiovascular function. Higher calcitriol concentrations are associated with reduced systolic blood pressure in hypertensive adults [3]. Low concentrations of calcidiol and calcitriol have been associated with adverse changes in several biomarkers, including higher renin concentration, glucose intolerance, albuminuria, and inflammation [4,5,6,7]. Production of calcitriol in the kidney is dependent on the blood concentration of parathyroid hormone (PTH), which promotes the synthesis of calcitriol by increasing the activity of 1-alpha hydroxylase in the kidneys. Conversely, increased calcium, phosphorus, and circulating fibroblast growth factor 23 (FGF-23) inhibit synthesis of calcitriol [8,9]. To maintain homeostasis, the regulation of calcitriol, PTH, calcium, and phosphorus concentrations in healthy individuals are interconnected in complex feedback loops [10]. The effect of dietary contents on calcidiol, and not calcitriol, has been studied previously [11,12,13,14].

The Dietary Approach to Stop Hypertension (DASH) trial was a randomized controlled feeding study that tested the effects on blood pressure of three types of dietary patterns—a control diet, a diet rich in fruits and vegetables (F&V), and the DASH diet [15]. Based on the results of this trial, the DASH diet is widely recommended as a healthful dietary pattern that substantially lowers blood pressure [16]. However, little is known about the effects of different dietary patterns, including DASH diet, on calcitriol and other aspects of mineral metabolism. In this context, we used data from the DASH trial to determine the effects of the three dietary patterns on blood concentrations of calcitriol, and the associated markers PTH, ionized calcium, and urinary excretion of calcium and phosphorus. In addition, we conducted supplemental analysis stratified by race and sex to check for interactions given known racial differences in bone mineral metabolism markers [17,18].

## 2. Methods

### 2.1. Study Design

The DASH trial was a multicenter, randomized feeding study designed to compare the effects of three dietary patterns on blood pressure: (1) a control diet; (2) a diet that is high in fruits and vegetables (F&V), but otherwise like the control diet; and (3) the DASH diet, that is rich in fruits, vegetables, dairy products and reduced in saturated fat, total fat, and cholesterol. Detailed methods have been published [19]. All subjects gave their informed consent for inclusion before they participated in the study. The study was conducted in accordance with the Declaration of Helsinki, and the protocol was approved by the Ethics Committee of Johns Hopkins School of Medicine #NA_00046895.

### 2.2. Participants

The DASH trial recruited adult men and women who were at least 22 years old. The study population was two-thirds minority, African-Americans. Participants had systolic blood pressure of less than 160 mm Hg and a diastolic blood pressure of 80 to 95 mm Hg (mean of six measurements) with normal renal function. Principal exclusion criteria were renal insufficiency, special dietary requirements, and use of antihypertensive medication, vitamins, mineral supplements, antacids containing magnesium or calcium, or other medications and dietary supplements that might affect blood pressure or micronutrient metabolism. Only supplement free, and anti-hypertensive-medication-free healthy men and women were enrolled. The study also excluded subjects who: were pregnant or lactating; had hyperlipidemia; had a cardiovascular event within the previous six months; had poorly controlled diabetes mellitus; had chronic diseases that might interfere with participation; had a body mass index (BMI) of more than 35; had an unwillingness to stop taking vitamin and mineral supplements or antacids containing magnesium or calcium; or had an alcoholic-beverage intake of more than 14 drinks (one standard drink) per week. Participant enrollment took place at five locations; (1) Baltimore, Maryland; (2) Durham, North Carolina; (3) Boston, Massachusetts; (4) Portland, Oregon; and (5) Baton Rouge, Louisiana. Enrollment occurred in waves across all sites during all seasons from September 1994 through January 1996.

### 2.3. Intervention

The DASH trial had three phases—screening, run-in, and intervention. The run-in phase was a three-week period of feeding in which all participants ate the control diet. The intervention phase was an eight-week period of feeding in which participants ate their assigned diet; no dietary supplements or any medications were allowed during the 11 weeks of feeding [19].

The control dietary pattern was typical of what many Americans eat—low in minerals (potassium, magnesium, and calcium) and high in cholesterol, saturated fat, and total fat. The F&V diet was high in magnesium, potassium, and fiber; however, it had the same amount of saturated fat, total fat, cholesterol, protein, and calcium as the control diet. The DASH dietary pattern had lower amounts of total fat, saturated fat, and cholesterol compared to the control diet, and higher amounts of potassium, calcium, magnesium, fiber, and protein (See Supplemental Table S1). Menus for each dietary intervention have been published [20]. Weight was tightly maintained by adjusting the caloric intake for each individual under each arm.

### 2.4. Measurements

Baseline concentrations of serum calcitriol (1,25-hydroxyvitamin D3), PTH, ionized calcium and 24-h urinary calcium and phosphorus concentrations were measured during the third and final week of the run-in period, throughout which all participants ate the control diet. Serum calcidiol was not measured because it was not included in the original study outcomes. After the 8-week intervention period, the study team measured these same concentrations to determine the end-of-intervention value. All sites followed standard procedures for blood and urine sample collection, processing, and analysis (including the 24-h urine collection). All measurements were done at the time of the original study. Adherence was measured through clinic rating, daily diary, run-in log, missed meals registry, attendance, missed foods and non-study food diary, and a case conference with compliance incentive activities.

### 2.5. Outcome

Our primary outcome for this paper was to measure the change, if any, from baseline to end-of-intervention in blood concentration of calcitriol measured in pg/mL. Changes in PTH concentrations (pg/mL), ionized calcium concentrations (mg/dL), and 24-h urine excretion of phosphorus (mg/24 h) and calcium (mg/24 h) were secondary outcomes. A common central laboratory performed all assays.

### 2.6. Statistical Analysis

Of the original 459 randomized participants, 334 individuals had complete data, i.e., baseline and end-of-intervention values for the outcomes of interest. Missing test results for some patients resulted in incomplete data sets for 125 participants. Baseline characteristics were comparable among the 334 individuals with a complete set of outcomes and the 125 without all outcomes (See Supplemental Table S2). We checked distributions, impact of influential outliers for each marker, and compared estimates with outliers dropped against the original estimates with outliers included. No meaningful difference was observed; therefore, our primary analysis used all data for the 334 individuals, including any outliers. We summarized characteristics of the study participants (*n*, %) or (mean ± SD) by randomization group.

For each individual, we calculated change by subtracting the end-of-intervention values from the end-of-run-in values. We tested differences in change for each outcome between each pair of diets in linear regression models after testing the assumptions of linear regression. Dependent variables were change in calcitriol, PTH, ionized calcium, and 24 h urinary calcium and phosphorus excretion. The primary independent variable was randomized group, i.e., dietary pattern. Dietary patterns modeled as dummy variables. To explore subgroup effects on each outcome, we stratified results by race and sex, and tested for corresponding interactions, i.e., a diet-race interaction and a diet-sex interaction. Due to the known relationship between geolocation and sun exposure, we also conducted sensitivity analyses to assess if the clinic’s site had any effect on the primary outcome. In addition, we stratified by race and sex to examine the effect of skin color between and within study outcomes. We reported the observed P-value since this was a supplemental analysis [21,22], and the adjusted *p*-value for multiple comparisons based on the number of pairwise comparisons using Bonferroni correction. We used STATA/IC 14.0 (StataCorp LP, 4905 Lakeway Drive, College Station, TX, USA) for all analyses.

## 3. Results

Table 1 displays the main baseline characteristics of our study participants, overall and by randomized dietary pattern. The average age for the participants was 45.7 ± 10.7 years, 46 percent were female, and 57 percent were African-Americans. The average weight (mean ± SD) was 82.8 ± 14.6 kg, and average BMI was 28.1 ± 3.9 kg/m^2^. Thirty-two percent of the participants were hypertensive at randomization. There were no statistically significant differences in characteristics among the three groups. The DASH study did not record the menopausal status of the women. However, only 27 percent of women were older than 51 years of age, which is the average age of menopause in the US. Participant’s adherence to the study diets was excellent; 95.5, 97.4, and 98.7 percent of participants completed the intervention phase in the control, F&V, and DASH groups, respectively.

### 3.1. Changes in Outcomes

Table 2 displays the within-group changes. Average (mean ± SD) baseline concentration of calcitriol among all participants was 37.8 ± 9.2 pg/mL. Serum concentrations of calcitriol decreased in the DASH group with a mean change of −4.0 ± 8.6 pg/mL, compared to −1.5 ± 7.8 pg/mL in the F&V group, and −0.7 ± 8.9 pg/mL in the control group. Table 3 shows the between-group differences. Compared to control, the DASH diet was associated with a statistically significant net reduction in calcitriol concentration (mean difference = −3.32 pg/mL (*p* = 0.004)). Calcitriol was also reduced in the DASH group compared to the F&V group (mean difference = −2.52 pg/mL, *p* = 0.026).

There were no within-group changes in serum concentration of ionized calcium and no significant between-group differences. Mean ± SD change in 24-h urinary excretion of calcium was −8.1 ± 56.4 mg/24 h in control, −48.7 ± 68.0 mg/24 h in F&V, and −4.4 ± 77.1 mg/24 h in DASH. Compared to F&V, the DASH group experienced an increase in calcium excretion (44.29 mg/24 h (*p* < 0.001)).

Mean ± SD change in 24-h urinary excretion of phosphorus was 61.7 ± 252.0 mg/24 h in control, −21.0 ± 269.4 mg/24 h in F&V, and 117.5 ± 272.7 mg/24 h in DASH. Compared to the F&V group, the DASH group experienced a statistically significant increase of 138.52 mg/24 h in urinary phosphorus excretion (*p* < 0.001).

### 3.2. Subgroup Effects

Supplemental Table S3 displays between-diet differences by race and sex.. There were no significant interactions between diet and either sex or race alone. However, the effect of the DASH diet on calcitriol, net of control, appeared to be more significant in black men even after adjusting for multiple comparison.

## 4. Discussion

In the DASH trial, a tightly controlled feeding study, the DASH diet significantly lowered blood calcitriol concentrations, and had no significant effect on PTH, ionized calcium, and urinary excretion of calcium and phosphorus net of the control diet. In subgroup analyses, the effects of the DASH diet on blood calcitriol concentrations appeared qualitatively greater in black men. The effects of the DASH diet on blood calcitriol concentrations may have resulted from the lower fat content (mainly saturated) of the DASH diet, which provided 25.6% kcal from fat compared to 35.7% kcal from fat in the control and F&V diets. The differences in dietary fat may have affected the absorption of vitamin D and indirectly affected the available pool of vitamin D to be converted to calcitriol. A previous study found that dietary fat content significantly affects vitamin D absorption. [12] However, blood concentrations of the calcidiol prohormone form of vitamin D were not measured in this study. Another explanation for reduction in calcitriol is skin color. In our stratified analysis, we detect a stronger signal of calcitriol reduction among African-Americans, and in particular among men (Supplemental Table S3). Prior studies showed higher PTH concentration and differences in calcium homeostasis in African-Americans [17,18]. In addition, skin with higher pigmentation is associated with reduced capacity to synthesize vitamin D [23,24,25,26]. This may suggest that individuals who are more dependent on dietary sources of vitamin D may be more likely to experience reduction in calcitriol with a diet such as DASH. However, it is unknown if this reduction in calcitriol is temporary, or if it will continue if those individuals continue to consume the DASH diet for a longer period.

PTH, ionized calcium, and phosphorus are involved in the regulation of vitamin D activation. While the DASH diet has a high calcium content, the serum levels and urine excretion of calcium were not changed significantly when compared to the control diet. Furthermore, there was no significant change in PTH concentration. However, there was a significant increase of phosphorus excretion after consuming the DASH diet within the DASH group, which may affect the activation of calcidiol to calcitriol via renal 25-Hydroxyvitamin D3 1-alpha-hydroxylase [27].

Associations of calcitriol on cardiovascular health have been reported in several studies. Calcitriol supplementation was associated with reduced blood pressure in a randomized controlled pilot and feasibility study among adults [3]. In a rodent study, higher calcitriol was associated with a decrease in endothelial-dependent contraction in rat aorta [28]. Findings from in vitro studies suggest that exposure to calcitriol (0.01–10 nmol/L) prevents vascular calcification in human vascular smooth muscle cells obtained from healthy human aortic tissues in a dose-dependent manner [29]. In addition, calcitriol may contribute to vascular repair by increasing the number of circulating angiogenic myeloid cells (AMC) [30], and decreases circulating pro-inflammatory cytokine TNFα [31]. Furthermore, supplementation with vitamin D3 has been shown to improve small proteins profiles in patients with congestive heart failure [31].

Evidence on elevated serum PTH, aside from the clinical conditions of abnormal serum calcium and vitamin D deficiency, has been associated with coronary heart disease [32] and cardiovascular mortality [33,34]. Hyperparathyroidism induces hypertrophy in cardiomyocytes and subsequently leads to development of left ventricular hypertrophy [35] and heart failure [36]. In our study, there was no significant change in PTH concentration, but a modest reduction in calcitriol. A possible explanation of why we did not detect any dynamic change in PTH may be due to the fact that PTH molecules are rapidly degraded by the liver with a half-life of 2–4 min, which later release non-(1-84) PTH and C-terminal fragment, and both have a half-life of 5–10 times the original molecules [37,38].

The clinical significance of this modest reduction of calcitriol seen with the DASH diet is uncertain. As documented previously, the DASH diet had favorable effects on bone and cardiovascular health. Specifically, the DASH diet reduced markers of bone turnover and had favorable changes in osteocalcin and C-terminal telopeptide of type I collagen [39].

Evidence on the relationship of ionized calcium with blood pressure and other aspects of cardiovascular health is inconsistent. In patients with untreated hypertension, serum ionized calcium was lower than those without hypertension [40]. In contrast, in a large cross-sectional study by Jorde et al., there was no association of ionized serum calcium with hypertension [41]. In our analysis, there was no change in serum ionized calcium concentration in any of the 3 diet groups.

Another interesting observation was the opposite effect of DASH and F&V diets on urinary excretion of calcium; the latter diet was associated with prominent reduction in calcium excretion compared to control, while DASH diet was associated with no significant difference in urinary calcium excretion compared to control. The observed significant increase in calcium excretion among DASH compared to F&V was due to the significant reduction in calcium excretion in F&V net of control, and not due to actual increase in excretion among DASH group. It is not clear why there was reduction in calcium excretion among the F&V group. Both the F&V and DASH diets were similarly high in fiber, a nutrient which lowers urinary excretion of calcium [42]. However, the DASH diet has a higher calcium content compared to the F&V diet (DASH = 1265 mg/day versus F&V = 450 mg/day).

Phosphorus has important roles in energy metabolism, signaling in cell metabolism, forming the phospholipid membrane, and protein synthesis [43]. High phosphorus concentration has been linked to vascular calcification and vascular stiffness through calcium phosphorus deposition on the blood vessels [44]. In our study, there was a significant increase in phosphorus excretion within the DASH diet group. Compared to F&V, DASH was associated with a significant increase in phosphorus excretion. This observation is not due to an actual increase in phosphorus excretion among the DASH group, but due to a substantial reduction of phosphorus excretion among the F&V group compared to the control group. There was no significant difference in net excretion of phosphorus among DASH compared to control group. In addition, DASH contains more phosphorus than either the F&V or control diets (DASH = 1729 mg/day, F&V = 1165 mg/day, Control = 1121 mg/day) [45].

Our study has limitations. The trial was originally designed to test the effects of the diets on blood pressure. Therefore, the sample size and trial design were geared towards that aim rather than biomarkers related to mineral metabolism. However, we reported the 95% confidence intervals on mean differences in outcomes between diets (Table 3). The study was also not powered for subgroup analysis, but we presented the 95% confidence intervals in the mean difference in outcomes by diets among subgroups by race and sex (Supplemental Table S3). Second, the entire feeding protocol lasted only 11 weeks, so the long-term effects of the diets on biomarkers are unknown. Third, we only had available concentrations of calcitriol, the active metabolite, not serum calcidiol concentrations for assessing vitamin D storage and deficiency/sufficiency states. Our trial also has several strengths. Feeding was conducted in a very controlled setting, in which no supplements or medications that affect mineral metabolism were allowed, and adherence was excellent. All blood and urine samples were collected and processed per standard procedures that were followed by all centers. Also, all samples were analyzed in the same central laboratory.

## 5. Conclusions

The DASH diet has been recommended as an effective dietary approach to lower blood pressure and improve cardiovascular health. In these analyses, we documented that the DASH diet was associated with modest reduction in calcitriol concentration among all participants, perhaps more so among African-American males. Further assessment of the long-term effects of DASH diet on markers of bone mineral metabolism and of bone health is warranted.

## Figures and Tables

**Table 1:** Baseline characteristics of the study participants, per dietary arm.

Characteristics	Total (*n* = 334)		Control Diet (*n* = 108)		Fruit and Vegetable Diet (*n* = 112)		DASH Diet‡ (*n* = 114)	
Age (Y) *	45.7	±10.7	45.0	±11.2	45.3	±1.8	44.3	±10.2
Female (*n*, %)	155	46	46	43	52	46	57	50
Female > 51 years (*n*, %)	42	27	11	23	15	28	16	28
Race								
Black (*n*, %)	192	57	62	57	64	57	66	58
Weight (kg)								
All	82.8	±14.6	82.9	±14.6	81.8	±14.0	83.4	±15.0
Male	88.0	±14.2	89.1	±12.7	86.1	±13.4	88.7	±16.7
Female	76.8	±12.5	74.8	±13.0	76.9	±13.2	78.2	±11.4
Body Mass Index, BMI (kg/m^2^) †								
All	28.1	±3.9	28.0	±3.7	28.0	±4.1	28.4	±4.0
Male	27.8	±3.7	28.0	±3.4	27.3	±3.5	28.1	±4.1
Female	28.5	±4.1	28.7	±3.8	29.0	±3.6	29.8	±4.0
Hypertensive (*n*, %)	105	31	28	35	37	33	30	26

‡ The “Dietary Approaches to Stop Hypertension” (DASH) diet. * Plus–minus values are means ± Standard Deviation (SD). † Body Mass Index (BMI) calculated by weight in kilograms divided by the square of the height in meters.

**Table 2:** Blood and urine markers at baseline (end-of-run-in), end-of-intervention, and difference by dietary pattern.

	Control Diet		Fruit and Vegetable Diet		DASH Diet	
	Mean	SD	Mean	SD	Mean	SD
Calcitriol (pg/mL)						
run-in	37.3	±10.1	37.6	±7.8	38.5	±9.7
intervention	36.6	±10.2	36.1	±9.6	34.5	±7.8
difference	−0.7	±8.9	−1.5	±7.8	−4.0	±8.6
Parathyroid Hormone, PTH (pg/mL)						
run-in	49.1	±20.1	44.7	±17.4	44.6	±17.7
intervention	48.3	±20.0	46.7	±19.4	43.6	±18.6
difference	−0.9	±13.9	1.9	±17.5	−1.0	±12.8
Ionized Calcium (mg/dL)						
run-in	5.2	±0.23	5.2	±0.23	5.2	±0.23
intervention	5.2	±0.21	5.2	±0.22	5.2	±0.23
difference	−0.0	±0.21	0.0	±0.26	−0.0	±0.23
24 h Urinary Calcium (mg/24 h)						
run-in	145.6	±75.8	155.4	±77.5	149.1	±80.1
intervention	137.5	±71.4	106.7	±66.8	144.7	±88.7
difference	−8.1	±56.4	−48.7	±68.0	−4.4	±77.1
24 h Urinary Phosphate (mg/24 h)						
run-in	683.0	±257.1	722.1	±243.7	717.8	±254.9
intervention	725.7	±236.6	702.1	±249.1	835.4	±333.3
difference	61.7	±252.0	−21.0	±269.4	117.5	±272.7

Average PTH concentration at baseline among all participants was 46.1 ± 18.5 pg/mL. The mean ± SD change in PTH concentrations from run-in to intervention within each arm is in Table 2. Compared to control or F&V diets, the DASH diet was associated with a non-significant reduction in PTH concentrations (Table 3).

**Table 3:** Comparisons of mean changes in calcitriol, PTH, ionized calcium, urinary calcium & urinary phosphorus between diets in all participants.

Markers	Δ-Δ	(95% Confidence Interval)	*p*
	Change in DASH Diet Minus Change in Control Diet		
Blood ‡			
Calcitriol	−3.32	(−3.05, −1.08)	0.004
PTH	−0.15	(−4.08, 3.78)	0.941
Ionized Calcium	−0.003	(−0.07, 0.06)	0.931
24-h urine §			
Urinary Calcium	3.67	(−14.29, 21.60)	0.689
Urinary Phosphate	55.86	(−14.33, 126.01)	0.180
Markers	Change in F&V Diet Minus Change in Control Diet		
Blood ‡			
Calcitriol	−0.81	(−3.05, 1.44)	0.481
PTH	2.80	(−1.14, 6.75)	0.163
Ionized Calcium	0.026	(−0.04, 0.08)	0.418
24-h urine §			
Urinary Calcium	−40.63	(−58.66, −22.61)	<0.001
Urinary Phosphate	−82.66	(−158.31, −12.01)	0.022
Markers	Change in DASH Diet Minus Change in F&V Diet		
Blood ‡			
Calcitriol	−2.52	(−4.73, −0.30)	0.026
PTH	−2.96	(−6.85, −0.94)	0.136
Ionized Calcium	−0.029	(−0.09, 0.03)	0.364
24-h urine §			
Urinary Calcium	44.29	(26.51, 62.07)	<0.001
Urinary Phosphate	138.52	(68.98, 208.06)	<0.001

‡ Calcitriol and parathyroid hormone measured in pg/mL, ionized calcium measured in mg/dL. § Urinary calcium and phosphorus measured in mg/24 h.

## Supplementary Materials

The following are available online at http://www.mdpi.com/2072-6643/10/3/367/s1, Figure S1: CONSORT flowchart, Figure S2: CONSORT 2010 Checklist of Randomized Trial ,Table S1:Nutrient Targets, Menu Analyses, And Average Daily Servings Of Foods, According To Diet, Table S2: Missing Data By Clinical Center, and Table S3: Comparisons Of Mean Changes In Calcitriol, Parathyroid Hormone, And Urinary Excretion Of Calcium Between Diets By Race And Sex.

## Availability of Data and Material

Data can be requested from National Heart, Lung, and Blood Institute by using BioLINCC: https://biolincc.nhlbi.nih.gov/home/. NCT00000544. Registered October 27, 1999 ‘retrospectively registered’ https://clinicaltrials.gov/ct2/show/study/NCT00000544?term=DASH&rank=81&show_desc=Y#desc.

## Additional Materials

Additional materials contain the nutrients analysis and number of servings within each diet, a summary of missing data, and further stratification analysis by race and sex. We also included the CONSORT diagram for the trial, and CONSORT checklist.

## Abbreviations

DASH	Dietary Approach to Stop Hypertension
PTH	Parathyroid Hormone
F&V	Fruit and Vegetable Diet
BMI	Body Mass Index
