# Supplementary material for: Effects of Different Dietary Interventions on Calcitriol, Parathyroid Hormone, Calcium, and Phosphorus: Results from the DASH Trial
Source: Nutrients. 2018 Mar 17;10(3):367. doi: 10.3390/nu10030367 (PMC5872785; doi:10.3390/nu10030367)
Supplement: Supplementary file 1 [file nutrients-10-00367-s001.pdf]

Online Supporting Material

Supplemental Materials: 3 Tables, 2 Figures.

SUPPLEMENTAL TABLE 1: NUTRIENT TARGETS, MENU ANALYSES, AND AVERAGE DAILY SERVINGS OF FOODS, ACCORDING TO DIET. *

	Control Diet		Fruits & Vegetable Diet		Combination Diet	
**Item**	NUTRIENT TARGET	MENU ANALYSIS	NUTRIENT TARGET	MENU ANALYSIS	NUTRIENT TARGET	MENU ANALYSIS
**Nutrients**						
Fat (% of total kcal)	37	35.7	37	35.7	27	25.6
Saturated	16	14.1	16	12.7	6	7
Monounsaturated	13	12.4	13	13.9	13	9.9
Polyunsaturated	8	6.2	8	7.3	8	6.8
Carbohydrates (% of total kcal)	48	50.5	48	49.2	55	56.5
Protein (% of total kcal)	15	13.8	15	15.1	18	17.9
Cholesterol (mg/day)	300	233	300	184	150	151
Fiber (g/day)	9	NA	31	NA	31	NA
Potassium (mg/day)	1700	1752	4700	4101	4700	4415
Magnesium (mg/day)	165	176	500	423	500	480
Calcium (mg/day)	450	443	450	534	1240	1265
Sodium (mg/day)	3000	3028	3000	2816	3000	2859
**Food groups (no. of servings/day)**						
Fruits and juices	1.6		5.2		5.2	
Vegetables	2		3.3		4.4	
Grains	8.2		6.9		7.5	
Low-fat dairy	0.1		0		2	
Regular-fat dairy	0.4		0.3		0.7	
Nuts, seeds, and legumes	0		0.6		0.7	
Beef, pork, and ham	1.5		1.8		0.5	
Poultry	0.8		0.4		0.6	
Fish	0.2		0.3		0.5	
Fat, oils, and salad dressing	5.8		5.3		2.5	
Snacks and sweets	4.1		1.4		0.7	

* Values are for diets designed to provide an energy level of 2100 kcal Values are for diets designed to provide an energy level of 2100 kcal

SUPPLEMENTAL TABLE 2: MISSING DATA BY CLINICAL CENTER

	Missing data				
	All	Baltimore	Baton Rouge	Boston	Durham
	N=459	n=92	n=114	n=124	n=129
**Parameters (Before)**					
24h Urinary Phosphate (mg/24h)	101	10	32	43	16
24h Urinary Calcium (mg/24h)	72	3	28	31	10
PTH (pg/ml)	6	1	0	4	1
Ionized Calcium (mg/dl)	5	1	0	4	0
Calcitriol (pg/ml)	7	1	0	6	0
**Parameters (After)**					
24h Urinary Phosphorus (mg/24h)	93	11	30	43	9
24h Urinary Calcium (mg/24h)	59	5	29	17	8
PTH (pg/ml)	33	1	4	25	3
Ionized Calcium (mg/dl)	33	1	4	25	3
Calcitriol (pg/ml)	37	4	4	26	3
**Missing one or more mean change**	125	14	41	49	21

SUPPLEMETAL TABLE 3. COMPARISONS OF MEAN CHANGES IN CALCITRIOL, PARATHYROID HORMONE, AND URINARY EXCRETION OF CALCIUM BETWEEN DIETS BY RACE AND SEX.

Markers	Change in DASH Diet Minus Change in Control Diet				Change in F &V Diet Minus Change in Control Diet				Change in DASH Diet Minus Change in F&V Diet			
	Δ-Δ	(95 CI)	P	Adjusted P‡	Δ-Δ	(95 CI)	P	Adjusted P‡	Δ-Δ	(95 CI)	P	Adjusted P‡
**CALCITRIOL (pg/ml)**												
**Black women**	-2.89	(-6.43, 0.65)	0.108	0.432	0.54	(-3.10, 4.18)	0.769	1.000	-3.43	(-6.98, 0.11)	0.057	0.228
**Black men**	-6.94	(-11.50, -2.37)	0.006	0.024	-6.32	(-10.76, -1.88)	0.006	0.024	-0.61	(-5.13, 3.91)	0.789	1.000
**non-Black women**	-2.41	(-12.54, 7.72)	0.633	1.000	4.74	(-5.58, 15.05)	0.358	1.000	-7.15	(-16.18, 1.88)	0.117	0.468
**non-Black men**	-0.65	(-4.11, 2.80)	0.708	1.000	0.26	(-3.16, 3.69)	0.878	1.000	-0.91	(-4.44, 2.61)	0.607	1.000
**PTH (pg/ml)**												
**Black women**	0.42	(-6.99, 7.84)	0.910	1.000	1.05	(-6.57, 8.67)	0.785	1.000	-0.62	(-8.05, 6.80)	0.867	1.000
**Black men**	-4.07	(-10.33, 2.18)	0.198	0.792	1.36	(-4.72, 7.45)	0.656	1.000	-5.44	(-11.63, 0.75)	0.084	0.336
**non-Black women**	-0.72	(-17.41, 15.97)	0.931	1.000	9.46	(-7.53, 26.45)	0.267	1.000	-10.17	(-25.05, 4.70)	0.174	0.696
**non-Black men**	2.76	(-3.52, 9.04)	0.385	1.000	3.45	(-2.77, 9.68)	0.274	1.000	-0.69	(-7.10, 5.71)	0.831	1.000
**Urinary Calcium (mg/24h)**												
**Black women**	-17.60	(-40.48, 5.27)	0.130	0.520	-56.19	(-79.68, -32.69)	<0.001	0.004	38.58	(15.69, 61.48)	0.001	0.004
**Black men**	27.06	(-14.85, 68.98)	0.202	0.808	-14.87	(-55.66, 25.91)	0.470	1.000	41.94	(0.44, 83.43)	0.048	0.192
**non-Black women**	5.29	(-68.12, 78.70)	0.885	1.000	-46.06	(-120.79, 28.68)	0.220	0.880	51.35	(-14.09, 116.78)	0.120	0.480
**non-Black men**	5.29	(-29.03, 39.62)	0.760	1.000	-43.52	(-77.55, -9.48)	0.013	0.052	48.81	(13.80, 83.82)	0.007	0.028

‡ Multiple comparisons adjusted P-Value (Bonferroni correction)

Online Supporting Material

Figure S1: CONSORT Chart

CONSORT Flow Diagram for DASH Trial

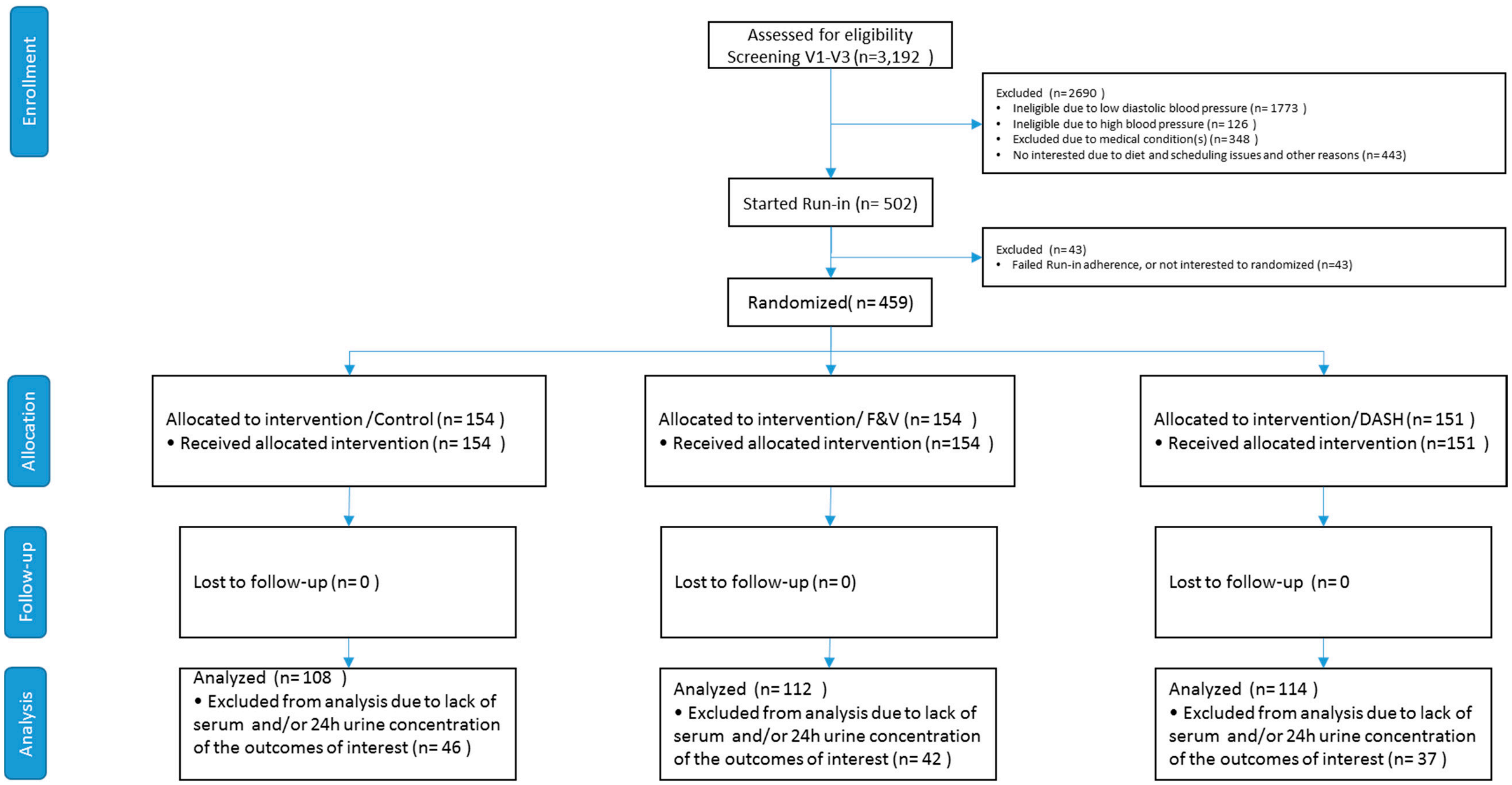

*Appel LJ, Vollmer WM, Obarzanek E, Aicher KM, Conlin PR, Kennedy BM, Charleston JB, Reams PM. Recruitment and baseline characteristics of participants in the Dietary Approaches to Stop Hypertension trial. DASH Collaborative Research Group. J Am Diet Assoc. 1999 Aug;99(8 Suppl):S69-75.

Figure S2: CONSORT 2010 Checklist of Randomized Trial*

Section/Topic	Item No	Checklist item	Reported on page No
**Title and abstract**			
	1a	Identification as a randomized trial in the title	1
	1b	Structured summary of trial design, methods, results, and conclusions (for specific guidance see CONSORT for abstracts)	3
**Introduction**			
Background and objectives	2a	Scientific background and explanation of rationale	4
	2b	Specific objectives or hypotheses	4-5
**Methods**			
Trial design	3a	Description of trial design (such as parallel, factorial) including allocation ratio	5
	3b	Important changes to methods after trial commencement (such as eligibility criteria), with reasons	5
Participants	4a	Eligibility criteria for participants	5
	4b	Settings and locations where the data were collected	5-6
Interventions	5	The interventions for each group with sufficient details to allow replication, including how and when they were actually administered	5
Outcomes	6a	Completely defined pre-specified primary and secondary outcome measures, including how and when they were assessed	6
	6b	Any changes to trial outcomes after the trial commenced, with reasons	N/A
Sample size	7a	How sample size was determined	6-7
	7b	When applicable, explanation of any interim analyses and stopping guidelines	N/A
Randomization:			
Sequence generation	8a	Method used to generate the random allocation sequence	5 (reference 14)
	8b	Type of randomization; details of any restriction (such as blocking and block size)	5 (reference 14)
Allocation concealment mechanism	9	Mechanism used to implement the random allocation sequence (such as sequentially numbered containers), describing any steps taken to conceal the sequence until interventions were assigned	5 (reference 14)
Implementation	10	Who generated the random allocation sequence, who enrolled participants, and who assigned participants to interventions	5 (reference 14)
Blinding	11a	If done, who was blinded after assignment to interventions (for example, participants, care providers, those assessing outcomes) and how	N/A
	11b	If relevant, description of the similarity of interventions	5-6

Statistical methods	12a	Statistical methods used to compare groups for primary and secondary outcomes	7
	12b	Methods for additional analyses, such as subgroup analyses and adjusted analyses	7
**Results**			
Participant flow (a diagram is strongly recommended)	13a	For each group, the numbers of participants who were randomly assigned, received intended treatment, and were analyzed for the primary outcome	7
	13b	For each group, losses and exclusions after randomization, together with reasons	7 + Suppleme ntal material
Recruitment	14a	Dates defining the periods of recruitment and follow-up	
	14b	Why the trial ended or was stopped	N/A
Baseline data	15	A table showing baseline demographic and clinical characteristics for each group	17
Numbers analyzed	16	For each group, number of participants (denominator) included in each analysis and whether the analysis was by original assigned groups	7
Outcomes and estimation	17a	For each primary and secondary outcome, results for each group, and the estimated effect size and its precision (such as 95% confidence interval)	8-9, 18-19
	17b	For binary outcomes, presentation of both absolute and relative effect sizes is recommended	N/A
Ancillary analyses	18	Results of any other analyses performed, including subgroup analyses and adjusted analyses, distinguishing pre-specified from exploratory	Suppleme ntal martials
Harms	19	All important harms or unintended effects in each group (for specific guidance see CONSORT for harms)	N/A
**Discussion**			
Limitations	20	Trial limitations, addressing sources of potential bias, imprecision, and, if relevant, multiplicity of analyses	12
Generalizability	21	Generalizability (external validity, applicability) of the trial findings	12
Interpretation	22	Interpretation consistent with results, balancing benefits and harms, and considering other relevant evidence	12-13
**Other information**			
Registration	23	Registration number and name of trial registry	2
Protocol	24	Where the full trial protocol can be accessed, if available	5
Funding	25	Sources of funding and other support (such as supply of drugs), role of funders	1
